# Resting-state electroencephalography based deep-learning for the detection of Parkinson’s disease

**DOI:** 10.1371/journal.pone.0263159

**Published:** 2022-02-24

**Authors:** Mohamed Shaban, Amy W. Amara

**Affiliations:** 1 Electrical and Computer Engineering, University of South Alabama, Mobile, AL, United States of America; 2 Neurology, University of Alabama at Birmingham, Birmingham, AL, United States of America; Kuwait College of Science and Technology, KUWAIT

## Abstract

Parkinson’s disease (PD) is one of the most serious and challenging neurodegenerative disorders to diagnose. Clinical diagnosis on observing motor symptoms is the gold standard, yet by this point nerve cells are degenerated resulting in a lower efficacy of therapeutic treatments. In this study, we introduce a deep-learning approach based on a recently-proposed 20-Layer Convolutional Neural Network (CNN) applied on the visual realization of the Wavelet domain of a resting-state EEG. The proposed approach was able to efficiently and accurately detect PD as well as distinguish subjects with PD on medications from subjects who are off medication. The gradient-weighted class activation mapping (Grad-CAM) was used to visualize the features based on which the approach provided the predictions. A significantly high accuracy, sensitivity, specificity, AUC, and Weighted Kappa Score up to 99.9% were achieved and the visualization of the regions in the Wavelet images that contributed to the deep-learning approach decisions was provided. The proposed framework can then serve as an effective computer-aided diagnostic tool that will support physicians and scientists in further understanding the nature of PD and providing an objective and confident opinion regarding the clinical diagnosis of the disease.

## Introduction

Parkinson’s disease (PD) which mostly affects the elderly population (age > 65 years old) is a neurodegenerative disorder that affects dopamine-producing neurons in the substantia nigra region of the brain [1]. Symptoms usually progress slowly over time ranging from mild tremor, changes in posture, walking and facial expressions to loss of balance, slowness of movements, frequent falls, stiffness, hallucinations and delusions, mood and sleep disorders and cognitive dysfunction. In advanced stages, the patient may be bedridden and requires around-the-clock care for all activities. In addition, cognitive impairment which is a non-motor complication of PD has been related to disease morbidity, significant burden on caregivers, social and working impairment, placement at long-term care facilities, and mortality [2]. According to Parkinson’s foundation, almost one million people suffer from the disease in the U.S. while it is estimated that 10 million individuals were diagnosed with the disease worldwide. In addition, 60,000 Americans are usually diagnosed with PD on an annual basis. It is also estimated that the health care cost for PD in the U.S. reaches $52 billion per year.

Evaluation of the motor and non-motor aspects of PD in the clinical setting is subjective and challenging [2]. Thus, there is a critical need to identify reliable biomarkers of PD that can be used by clinicians to monitor disease progression and response to therapeutic treatments. In addition, the development of early diagnosis and screening tools that may be able to identify subjects with high risk to develop prodromal PD will potentially improve the efficiency of administered therapeutic treatments and therefore eventually slow down the progression of the disease.

Electroencephalography (EEG) is considered as a potential diagnostic modality that may identify unique features of PD. Using this modality, researchers observed that beta and gamma power in PD are reduced [3, 4]. Further, persons with PD exhibit a slowing of resting-state oscillatory brain activity [5, 6] and changes in phase-amplitude coupling when compared to healthy controls (HC) [7, 8].

Machine and deep learning techniques (MDL) [9–17] can provide efficient solutions for various medical applications [18]. Several MDL approaches were introduced as an alternative to standard spectral analysis methods to identify the unique features of EEG and predict PD with an accuracy that ranges from 88% to 99.7% [19–28]. The aforementioned techniques either used MDL directly on the EEG data such as in [23–25] or transformed the EEG signal into a time-frequency representation (TFR) followed with feature extraction and classification using MDL as in [26] or TFR transformation and MDL for both feature extraction and classification [27, 28]. However, in most cases, the proposed methods exhibit limited accuracy as in [23–25] without a thorough assessment for the robustness and reliability of the methods based on the weighted Kappa score. In addition, features detected by the models were not interpreted and the basis for the provided predictions were not emphasized.

In this paper, a novel deep-learning approach was introduced that exploits the Wavelet domain of a resting-state EEG time-series in order to classify subjects into HC and PD in order to support the clinical diagnosis of the disease. The contributions of this study can be summarized as follows:

1. This is the first time where a continuous wavelet-based deep learning approach was utilized to exploit the resting-state EEG for subjects with a confirmed diagnosis of PD offering a precise screening for the subjects (i.e., accuracy, sensitivity, specificity, Area Under Curve (AUC) and Weighted Kappa Score up to 99.9%) to support the clinical diagnosis of the disease. The achieved performance is the highest achieved among the recent state-of-the-art deep-learning applications on EEG for PD detection and diagnosis [23–28].
2. The deep-learning approach was also deployed for the first time to distinguish subjects that are OFF medications from subjects that are ON medications in order to understand the changes due to the therapeutic treatment initiation. Such experiment was not investigated in [23–28].
3. A three-class challenge was addressed, where subjects with and without medications and HC were identified from resting-state EEG achieving the highest possible accuracy (i.e., 99.6%) as compared to [23–28] including the deep learning approach [28] that was deployed for the three-class application with an accuracy of 99.46%.
4. The feature and class discriminative maps identified at the final convolutional layer of this approach were visualized using the Grad-CAM method [50] offering further insights on the features attributed to the disease as well as treatment initiation. The observations of the discriminative features of the Wavelet domain for PD (OFF and ON medications) were reported as well.

## Related work

EEG is a non-invasive modality that has gained traction to obtain mechanistic and granular information about brain activities and diseases including PD. Several studies have explored electrophysiological indices based upon spectral power in different frequency bands in both EEG, and magnetoencephalography (MEG) (e.g., delta [1–4 Hz], theta [4–8 Hz], alpha [8–12 Hz], beta [12–35 Hz], and gamma [35–45 Hz] waves) for PD patients [3–8].

In [3], a Fast Fourier Transform (FFT) was used on EEG data to show reduced power in the beta band with no detectable change in the theta, and alpha bands. In [4], it was reported that non-demented PD patients showed slowing of resting state oscillatory brain activity compared to controls. An increase in the theta power, and a decrease in beta, and gamma powers were also observed. However, demented PD patients showed an increase in delta, and theta powers, and a decrease in alpha, beta, and gamma powers. In [5], FFT was also applied to EEG data. An increase in the power in the theta band, and slowing in predominant frequencies for non-demented PD patients compared to controls was observed. An increase in the delta band activity was also detected among demented PD patients. A consistent increase in power in the delta, and theta bands, and a decrease in power in the alpha band was observed for demented PD patients [6]. Phase amplitude coupling (PAC) which is the coupling of the beta phase to the gamma amplitude was found to be elevated in the PD population as compared to subjects without movement disorders [7, 8].

The promising intersection of EEG data with MDL techniques demonstrates that MDL can precisely identify disease features or risks, and thus may have utility for screening patients. Vanegas et al. proposed three MDL frameworks: deploying Extra Tree Classifier, Linear Regression, and Decision Tree to identify EEG based biomarkers of PD with an AUC of 99.4%, 94.9%, and 86.2% respectively [19]. Oh et al. proposed a 13-layer Convolutional Neural Network (CNN) on resting-state EEG to detect de novo PD which achieved an accuracy of 88.25% [20]. In [21] Wagh et al., an 8-layer graph-CNN was proposed to classify various neurological diseases including PD with an accuracy of 85%. Koch et al. proposed a Random Forest Classifier to detect PD based on both clinical and automated features from EEG data with an AUC of 91% [22].

In [23], Shi et al., proposed two hybrid models including two-dimensional CNN-Recurrent Neural Networks (RNN) and three-dimensional CNN-RNN, where the former model achieved an accuracy of 82.89% for detecting PD. In [24], Lee et al. proposed a hybrid model using CNN and Long-Short Term Memory (LSTM) to exploit both the spatial and temporal features of EEG respectively with an accuracy of 96.9% for differentiating PD from HC. The model learns representations closely related to clinical features such as disease severity and dopaminergic levels. Our prior work has adopted an ANN based framework applied on three spatial channels of EEG including Oz, P8 and FC2 to screen subjects into PD and controls with an accuracy of 98%, sensitivity of 97%, and specificity of 100% [25]. Khare et al. have introduced the use of different machine learning methods including the Least Squares Support Vector Machine (LSSVM) on five different features extracted from the tunable Q-factor wavelet transform (TQWT) of a resting-state EEG dataset to discriminate HC from PD subjects with and without medications at an accuracy of 96% and 97.7% [26]. Khare et al. have also recently applied a 2D-CNN on the smoothed pseudo-Wigner Ville distribution (SPWVD) transformation of two EEG datasets with a validation accuracy of 99.9% and 100% respectively [27]. Loh et al. have also applied a 2D-CNN on the Gabor transform of a resting-state EEG dataset in order to classify subjects into HC and PD with and without medications at an accuracy of 99.5% [28]. In [29], Murugappan et al. have introduced the use of several machine learning algorithms including k-nearest neighbor, random forest, decision tree and extreme learning machine to classify the emotional state of PD patients into happiness, sadness, fear, anger, surprise and disgust based upon the features extracted from the low-pass and high-pass of the TQWT of EEG signals. The proposed approach achieved an accuracy, sensitivity and specificity of 94%, 96% and 82% for PD detection and identification.

MRI is usually considered by neurologists for the clinical diagnosis of neurological diseases. Zhang et al. proposed a novel approach for screening de novo PD using ResNet (i.e., a deep CNN) with broad views using two-view MRI data (i.e., AXI and SAG) with an accuracy of 76.46% [30]. Ramirez et al. introduced three fully convolutional Autoencoder models to detect de novo PD in Diffusion Tensor Imaging (DTI) MRI data with a best AUC of ROC of 77% [31]. Prasuhn et al. also proposed a binary Support Vector Machine (SVM) and used Multiple-Kernal Learning (MKL) to detect PD in DTI with no more than 60% specificity [32]. Their findings suggested that DTI-based analysis is not useful for correct differentiation of subjects with PD from HC.

Speech analysis has also been used to detect and distinguish subjects with PD from HC. Frid et al. used CNN on raw speech to distinguish between various stages of PD with a high accuracy [33]. In [34], SVM, and random forests were introduced to classify speech signals of 33 PD patients and 10 controls with an accuracy of 99%, achieved using 10 dysphonia features. Rasheed et al. proposed a Back Propagation Algorithm with Variable Adaptive Momentum (BPVAM) for detection of de novo PD applied on speech data with an accuracy of 97.5% [35]. Gunduz proposed two CNNs based on vocal data features to classify PD with an accuracy of 84.5%, and 86.8% respectively [36]. Karabayir et al. proposed Light Gradient Boosting (GB) and Extreme GB to detect PD from vocal data with an accuracy of 84.1%, and 81.6% respectively [37]. Zhang et al. introduced stack autoencoders (SAE) for diagnosing PD over the telephone where personal information and vocal data are fed to the machine learning algorithm to analyze the speech records [38].

In addition, wearable sensors have been adopted for collecting data related to PD. Moon et al. proposed a machine learning approach based on neural networks to distinguish between essential tremor (ET) and PD which have similarities in clinical characteristics including movement and gait [39]. El Maachi et al. proposed a deep neural network consisting of 18 parallel CNNs followed with a fully connected network to exploit relevant gait information and diagnose PD with an accuracy of 98.7% [40]. Zeng et al. introduced a mathematical model for the gait dynamics of subjects that determines output results by approximating the gait dynamics via radial basis achieving an overall accuracy of 96.39% [41].

Muniz et al. used logistic regression, probabilistic neural network (PNN), and SVM in diagnosing PD when ground reaction force (GRF) was considered as in input and the effectiveness of PD treatments were compared [42]. Pfister et al. proposed a CNN to classify PD into three movement states (OFF, ON, and dyskinesia (DYS) motor states) using data from wearable sensors achieving a low accuracy of 65% [43]. Drotar et al. proposed the use of feature selection and SVM methods to differentiate between 37 PD patients and 38 controls based on handwriting movements with an accuracy of 84% and 78% respectively [44]. Eskofier et al. compared the use of machine learning methods including SVM and k-nearest neighbors’ algorithms with CNN to classify inertial measurement units’ data obtained using wearable sensors attached to the right and left limbs of ten idiopathic PD patients. CNN outperformed the machine learning methods by at least 4.6% [45].

Ricci et al. used Naïve Bayes, SVM, and k-NN to detect de novo PD from wearable sensor data with SVM achieving the highest accuracy of 95% [46]. Talitckii et al. proposed using several machine learning approaches to differentiate PD from other neurological disorders characterized by motor differences using wearable sensors that would help minimize misdiagnosis of PD with the best accuracy of 85% [47]. Pereira et al. collected handwriting data for HC and PD and created the "HandPD" dataset [48]. Naive Bayes, optimum-path forest, and SVM were used for classification where the Naive Bayes achieved the highest accuracy of 78.9%. Further, Pereira et al. developed a CNN architecture to classify the "HandPD" dataset into one of two categories (i.e., PD or Controls) with an improved accuracy compared to the machine learning methods used in this task [49]. Moreover, Pereira et al. introduced CNN architectures for classifying handwriting dynamics obtained from a smart pen equipped with a series of sensors for 224 PD patients and 84 controls [50]. In [51], the author used a fine-tuned pre-trained VGG-19 to differentiate between PD and controls based on wave and spiral handwriting datasets. The proposed model achieved an elevated accuracy and sensitivity of over 88% and 86% respectively.

Although the prior work has addressed the use of MDL on EEG as well as other modalities, the classification accuracy, sensitivity and specificity in the majority of the methods are still limited with no explanation for the disease features detected by the proposed methods. In this paper, we introduce an efficient deep learning approach with an accuracy, sensitivity and specificity that almost reached 99.9% for classifying subjects into HC, PD with and without treatments. Further, a visualization and explanation of the classification results were provided that may potentially assist future clinical studies in further understanding the characteristics and biomarkers of the disease. We believe that a reliable and successful computer-aided diagnostic tool based upon machine or deep learning should be characterized by sensitive and accurate predictions outperforming the human or expert graders where the experts’ diagnosis accuracy was estimated to be 83.9% which was deemed unsatisfactory [56] as well as providing an explanation for the attained prediction to support the clinical diagnosis and elevate the confidence in such predictions.

## Materials and methods

### Dataset

The EEG dataset analyzed in this study was acquired at the Aron lab at the University of California at San Diego and further curated by the Swann lab at the University of Oregon. The dataset is on OpenNeuro where the latest version of 1.0.4 was published in January 2021 [52].

The dataset includes EEG samples for fifteen right-handed PD patients (eight females, mean age 62.6 ± 8.3 years), and sixteen matched HC (nine females, 63.5 ± 9.6 years) based on age, gender, and handedness. All PD patients have either mild or moderate PD. The patients were recruited from Scripps Clinic in La Jolla, California, and HC were volunteers from the local community.

**Fig 1:**
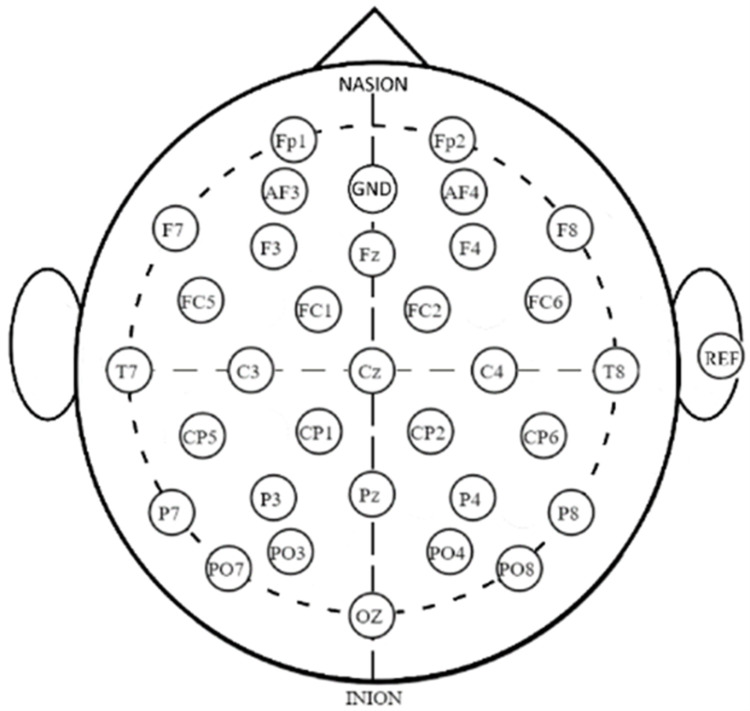
Locations of 32 electrodes of EEG.

EEG data were initially created in a Brain Imaging Data Structure (BIDS) format. Using Matlab EEGLAB tool, the data were then inserted into forty-six Excel files for the fifteen PD subjects (ON and OFF medication) and the sixteen HC. ON medication EEG data were recorded for the subjects who received treatments including Levodopa equivalent dose (three times/day). The EEG data were acquired using thirty-two standard electrodes at a sampling rate of 512 S/s within 1.9 to 2 minutes. The locations of the 32-channel EEG electrodes are shown in Fig 1.

### Proposed wavelet-based deep learning framework

**Fig 2:**
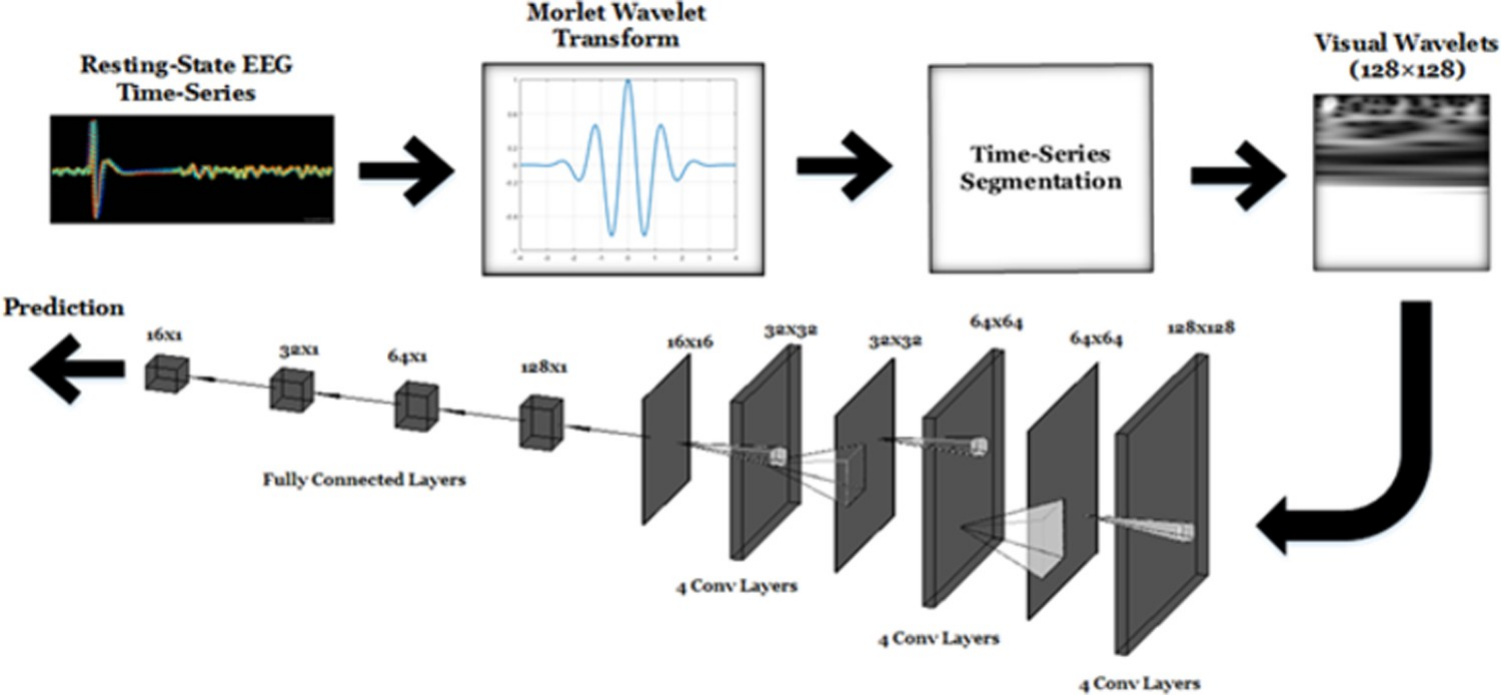
Proposed deep-learning approach.

The proposed framework consists of three main operations: Continuous Wavelet Transform (CWT), Time-Series Segmentation, and deep learning using CNN. The block diagram of this approach is illustrated in Fig 2.

The CWT was applied on the EEG signal *x*_*i*_*(t)* which is recorded at the *i*^th^ electrode to transform the EEG signal from the time-domain into the scale-time domain. The CWT operation is defined as follows:

$$X_{i}(s,\tau )=\frac{1}{\sqrt{s}}\int_{0}^{\infty }x_{i}(t)\psi (\frac{t-\tau }{s})dt$$ (1)

where *Ψ* is the Morlet analysis Wavelet, *τ* and *s* are the time shift and the scale of the Wavelet respectively. The scale of the Morlet Wavelet transform is the reciprocal of Fourier frequency where larger scales represent lower frequencies and vice versa [53]. In addition, the magnitudes of the Wavelet transform |*X_i_*(*s,τ*)| were generated and scaled for each subject and at each electrode *i*. This provided two-dimensional (i.e., 138×96,768 for HC and 138×97,792 for PD) matrices where the first dimension represents the scale and the second dimension represents time.

**Fig 3:**
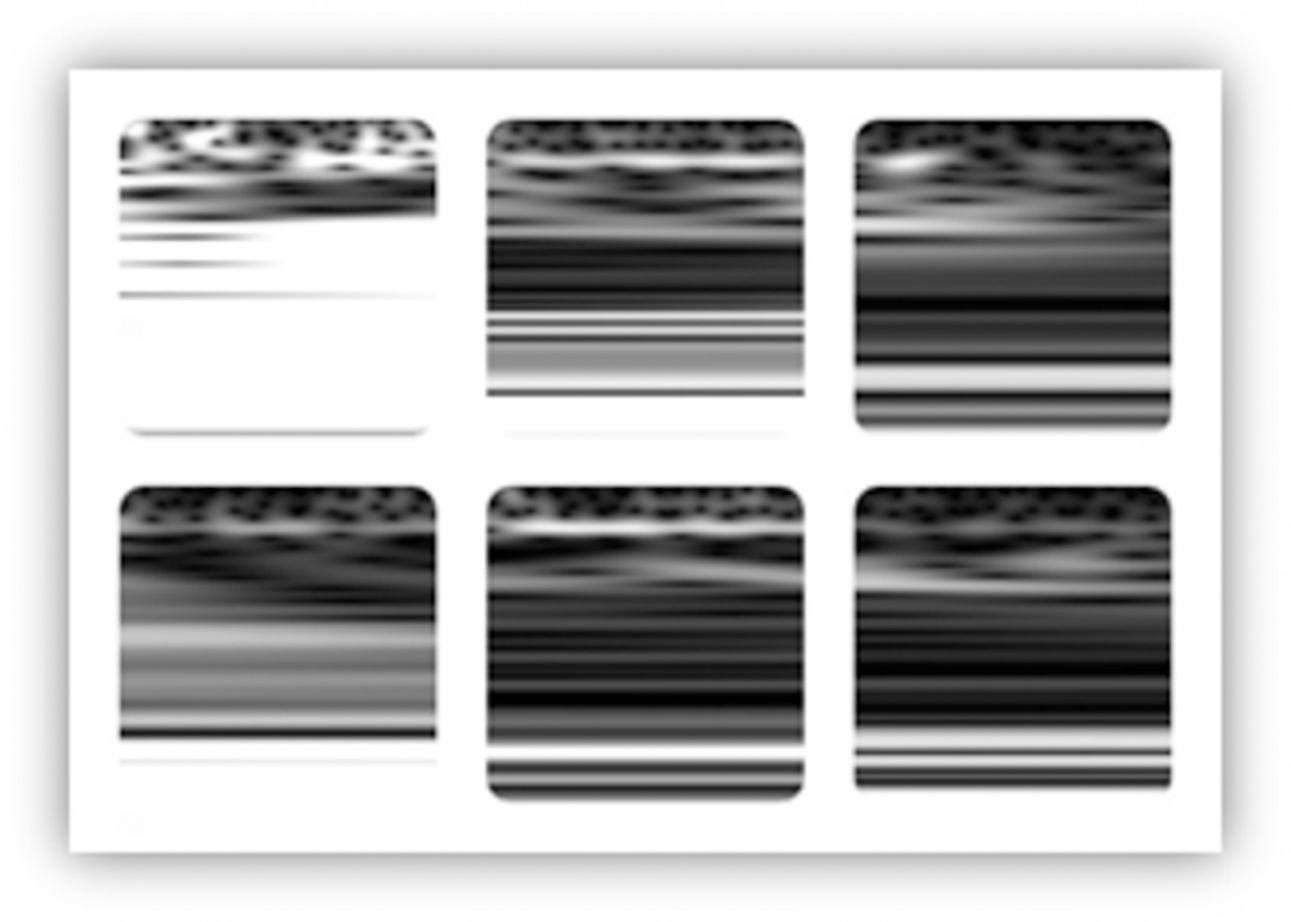
EEG wavelet images for HC (first column), PD OFF medication (second column) and PD ON medication (third column) for channels Fp1 and CP5. Channel Fp1 is represented in the first row and channel CP5 is represented in the second row.

Although the number of subjects used in this study is limited (i.e., 29 subjects), the application of a time-series segmentation of the aforementioned two-dimensional signals into 128×128 samples (The lowest 128 scales were selected out of 138 scales) provided sufficient number of training data samples for the deep learning approach to successfully identify the class of interest. Gray-scale scalograms of the segmented wavelets were then generated and provided for a second stage of a CNN based deep-learning model. Examples of EEG Wavelets for HC and PD recorded by the **Fp1** and **CP5** electrodes are shown in Fig 3.

The deep learning approach was used to achieve the following tasks. 1. Distinguishing HC from subjects with PD who do not receive treatments; 2. Classifying subjects into PD without and with therapeutic intervention; 3. Discriminating HC from PD OFF medication and PD ON medication. The deep learning approach adopted in this study uses an efficient CNN that was recently proposed by Shaban et al. and validated on a computer vision application (i.e., detection of oil spill from satellite aperture radar images) [54]. The components of the CNN are listed in Table 1.

**Table 1:** CNN structure.

Layer	Layer Size	Layer Depth	No. of Layers	Layer Properties
Input	128×128	1	1	-
Convolutional / ReLU	11×11	32	4	Same Padding
MaxPooling	2x2	32	1	No Padding
Convolutional / ReLU	9×9	64	4	Same Padding
MaxPooling	2x2	64	1	No Padding
Convolutional / ReLU	7×7	128	4	Same Padding
MaxPooling	2×2	128	1	No Padding
Fully Connected / ReLU	128, 64, 32, 16	1	4	-
Fully Connected / SoftMax	2	1	1	-

The deep learning network consists of 20 layers of convolutions, rectified linear units (ReLU), and maximum pooling (MaxPooling). The SoftMax probabilities of the network outputs were calculated and the cross entropy loss was estimated and minimized using the gradient stochastic descent. Finally, the probabilities calculated using the SoftMax function were then compared with an appropriate threshold providing a classification decision (i.e., 0 for HC, 1 for PD OFF medication and 2 for PD ON medication).

### Performance evaluation

Both four-fold and ten-fold cross-validation accuracy, sensitivity, and specificity were estimated in this study to evaluate the performance of the deep-learning approach to classify and identify HC, PD OFF medication, and PD ON medication. The aforementioned performance measures are defined as follows:

$$\mathrm{Accuracy}=\frac{TP+TN}{TP+TN+FP+FN}$$ (2)

$$\mathrm{Sensitivity}=\frac{TP}{TP+FN}$$ (3)

$$\mathrm{Specificity}=\frac{TN}{TN+FP}$$ (4)

where TP, FP, TN, and FN are the number of gray-scale images that were predicted as true positive (i.e., ground truth: PD and prediction: PD), false positive (i.e., ground truth: HC and prediction: PD), true negative (i.e., ground truth: HC and prediction: HC), and false negative (i.e., ground truth: PD and prediction: HC) respectively. The AUC of the Receiver Operating Characteristic Curve (ROC) was measured to assess the separability of the classifier. To ensure that the agreements among the predicted and the ground truth labels were not random, the Quadratic Weighted Kappa score (K) was used to evaluate the performance of the deep-learning method as follows:

$$K=1-\frac{\sum_{i=0}^{L}\sum_{j=0}^{L}w(i,j)c(i,j)}{\sum_{i=0}^{L}\sum_{j=0}^{L}w(i,j)p(i,j)}$$ (5)

where *L* is the number of classes which the images belong to (i.e. *L* = 2 or *L = 3*), *c (i*,*j)* and *w (i*,*j)* are the elements of the normalized confusion matric *C* and the weight matrix respectively where

w(i,j)=(i−j) (6)

Further, *p (i*,*j)* is an entry of the normalized outer product of the two normalized histograms for predicted and actual labels. To further understand the discriminative nature of the three classes (i.e., HC or PD (OFF medication) or PD (ON medication)), the Gradient-Weighted Class Activation Mapping (Grad-CAM) was applied on the gray-scale Wavelet transform images to visualize the feature maps of the last non-fully connected layer (i.e., layer no. 16, which is the last max-pooling and spatial filtering applied on the images) [55].

In the Grad-CAM method, the global average pooling is applied on the gradient of the class score *y^c^* with respect to the 128-feature maps *A_k_* of the 16^th^ layer where *k* is the feature map index as follows:

wkc=1S∑j∑j∂yc/∂Aijk (7)

where $w_{k}^{c}$ represents the significance of the *k*^*th*^ feature map of the 16^th^ layer in discriminating the class *c* in the image from other classes, *S* is the size of the feature maps, and $A_{ij}^{k}$are the pixel values of the *k*^*th*^ feature map of the 16^th^ layer. Further, the weights are multiplied with the 128-feature maps and rectified using a ReLU layer to generate the heat feature maps (i.e., class discriminative maps) as follows:

LGrad−CAMc=ReLU(∑kwkcAk) (8)

The Grad-CAM method was selected due to the simplicity of calculating the weights $w_{k}^{c}$ without the need for retraining the model. Further, the generated heat maps will pinpoint the regions within the wavelet images that the deep-learning approach considers to successfully classify as HC or PD (OFF medication) or PD (ON medication).

## Experimental study

Four different experiments were conducted using the deep-learning approach as follows:

### HC versus subjects with PD (OFF medication)

The objective of the first experiment is to classify subjects into HC and PD (OFF medication). This will support the clinician’s decision for screening subjects based on the recorded EEG.

In this experiment, the Morlet Wavelet transform was applied on the EEG time-series signals for the 16 HC and 15 PD (OFF medication) generating 24,264 gray-scale images of a dimension 128×128×1 at each of the 32 spatial channels. A total of 12,260 images were labelled as HC while 12,004 were related to PD. Four different channels (i.e., Fp1, FC1, CP5, and Fp2) were randomly selected for the analysis. The 4-fold and 10-fold cross-validation methods were deployed to evaluate the performance of the model where the training and validation images were separated based on the patient ID. Training images were then grouped into patches of 50 and the back propagation algorithm was executed at a learning rate of 10^−5^ for 40 epochs. Tables 2 and 3 show the 4-fold and 10-fold cross-validation training, cross-validation accuracy, sensitivity, specificity, weighted Kappa score, and AUC respectively for four different channels.

**Table 2:** 4-Fold cross validation results.

Channel	(Fp1)	(FC1)	(CP5)	(Fp2)
**Training Accuracy**	100%	100%	100%	100%
**Validation Accuracy**	98.6%	99.7%	99.9%	98.9%
**Sensitivity**	98.9%	99.8%	99.9%	99.1%
**Specificity**	98.3%	99.6%	99.9%	98.8%
**Weighted Kappa**	0.97	0.99	0.99	0.98
**AUC**	0.99	0.99	0.99	0.99

**Table 3:** 10-Fold cross validation results.

Channel	(Fp1)	(FC1)	(CP5)	(Fp2)
**Training Accuracy**	100%	100%	100%	100%
**Validation Accuracy**	98.7%	99.8%	99.9%	98.9%
**Sensitivity**	98.6%	99.8%	99.9%	98.8%
**Specificity**	98.7%	99.8%	99.9%	98.9%
**Weighted Kappa**	0.97	0.99	0.99	0.97
**AUC**	0.97	0.98	0.99	0.99

**Fig 4:**
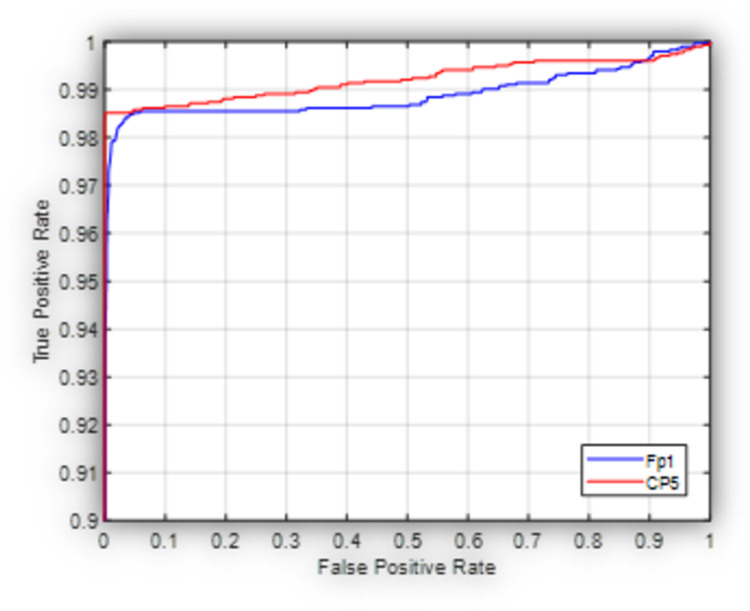
Receiver operating characteristic curve for the proposed model used to classify subjects into HC, and PD (OFF medication) at Fp1 and CP5.

Fig 4 shows the ROC graph for the proposed model in this case scenario. It is obvious that the proposed model performs better when applied on the CP5 channel as compared with Fp1 channel where the measured AUC is 0.99 and 0.97 respectively.

The confusion matrix for the 4-fold cross validation experiments at **Fp1, CP5** and **Fp2** channels is shown in Table 4. Indeed, Table 4 shows that the deep-learning approach achieves a minimal classification error ranging from 5 to 57 misclassified wavelet images out of 6066 images. Also, fewer PD images were misclassified as HC (i.e., less false negative rate) as compared to the false positive rate indicating that the approach can serve as a powerful pre-screening method that can be used prior to the application of the standard clinical tests.

**Table 4:** Confusion matrix for the deep-learning approach (4-fold cross validation).

Fp1		
	HC	PD
**HC**	3042	38
**PD**	19	2967
**CP5**		
	**HC**	**PD**
**HC**	3077	3
**PD**	2	2984
**Fp2**		
	**HC**	**PD**
**HC**	3021	37
**PD**	18	2990

### Subjects with PD (OFF medication) versus subjects with PD (ON medication)

In this study, the objective is to identify PD subjects who are ON and OFF medication. The ability of the approach to discriminate PD patients ON and OFF medication based on the resting-state EEG may support future studies to assess the efficacy of these treatments and monitor the changes in the EEG brain waves. In this case, the Wavelet transform is applied on the resting-state EEG corresponding to 15 subjects with PD (ON and OFF medication). The CNN was also applied with the same training and validation setup described in the previous sub-section. Table 6 presents the 4-fold cross-validation performance of the approach.

**Fig 5:**
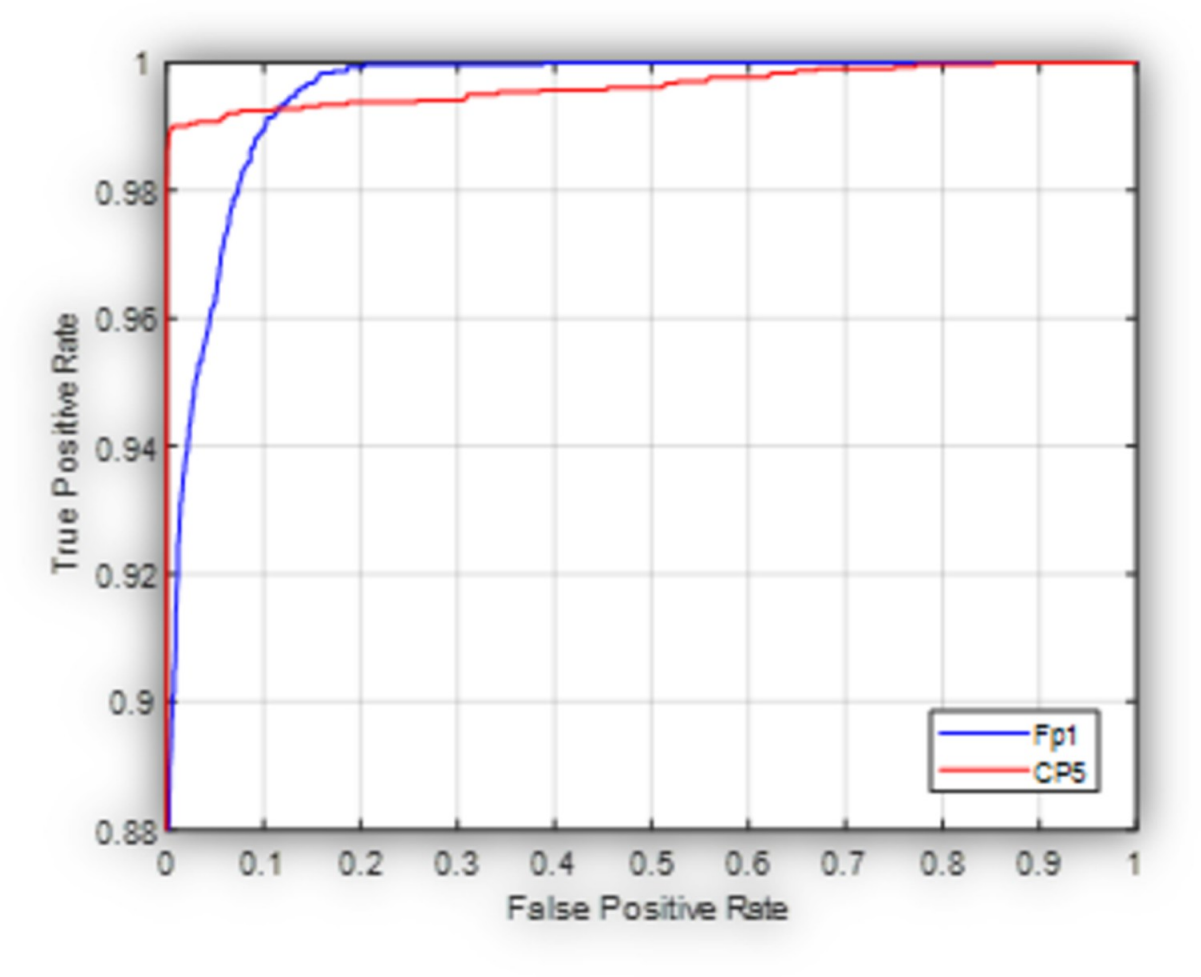
Receiver operating characteristic curve for the proposed model used to classify subjects into PD (OFF medication) and PD (ON medication) at Fp1 and CP5.

The reported results in Table 5 shows a promising use of the approach to identify PD with and without treatment at an accuracy up to 99.8% at **CP5**. Fig 5 shows the ROC graph for the proposed framework when used to discriminate subjects who are not receiving medical treatments from subjects on medications.

**Table 5:** 4-Fold cross validation results.

Channel	(Fp1)	(FC1)	(CP5)	(Fp2)
**Training Accuracy**	99.7%	99.7%	99.9%	99.9%
**Validation Accuracy**	96.3%	99.3%	99.8%	98%
**Sensitivity**	96.6%	99.3%	99.7%	97.8%
**Specificity**	95.9%	99.4%	99.8%	98.2%
**Weighted Kappa**	0.93	0.99	0.99	0.96
**AUC**	0.99	0.99	0.99	0.99

It is clear that the at a relatively low false alarm probability (i.e., false positive rate), the proposed approach is slightly sensitive to identify patients who are ON medications as well as who are OFF medication at CP5 as compared to Fp1 with almost the same AUC of 0.99 at both channels. This may assist clinical studies to monitor the effects of therapeutic treatments initiation and verify whether PD subjects receiving therapeutic treatments may or may not exhibit the same EEG changes as subjects not receiving the treatments. A potentially even more exciting use of this would be to test new therapeutic interventions to see if the experimental treatment causes the same EEG changes that known effective PD medications induce to predict potential for clinical efficacy.

### HC versus subjects with PD (OFF medication) and subjects with PD (ON medication)

In this experiment, we investigate the efficacy of the deep-learning approach when applied over the three cohorts (i.e., HC, PD (ON Medication) and PD (OFF Medication)). This will show the scalability of the approach over a multi-class problem when subjects can be directly screened into HC or PD with and without treatments from the resting-state EEG. Table 6 presents the 4-fold cross validation performance metrics in this scenario.

**Table 6:** 4-Fold cross validation results.

Channel	(Fp1)	(FC1)	(CP5)	(Fp2)
**Training Accuracy**	99.6%	99.9%	100%	99.6%
**Validation Accuracy**	95.5%	99.6%	99.6	96.2%
**Sensitivity**	95.4%	99.6%	99.6%	96.2%
**Specificity**	97.7%	99.8%	99.8%	98.1%
**Weighted Kappa**	0.94	0.99	0.99	0.95
**AUC**	0.98	0.99	0.99	0.99

The approach proved to maintain a significantly high 4-fold cross-validation accuracy, sensitivity and specificity up to 99.6% at **CP5**. The weighted Kappa score was ranging from 0.94 to 0.99 showing the robustness of the approach and offering an evidence on the reliability of the performance recorded at the four different channels. In addition, the performance of the approach has slightly dropped to 95.5% and 96.2% at **Fp1** and **Fp2** respectively as compared to the respective value at the central electrode **CP5**. This may be attributed to proximity of the frontal electrodes to eyes which make those electrodes more prone to eye movements and limit the classifier performance.

**Fig 6:**
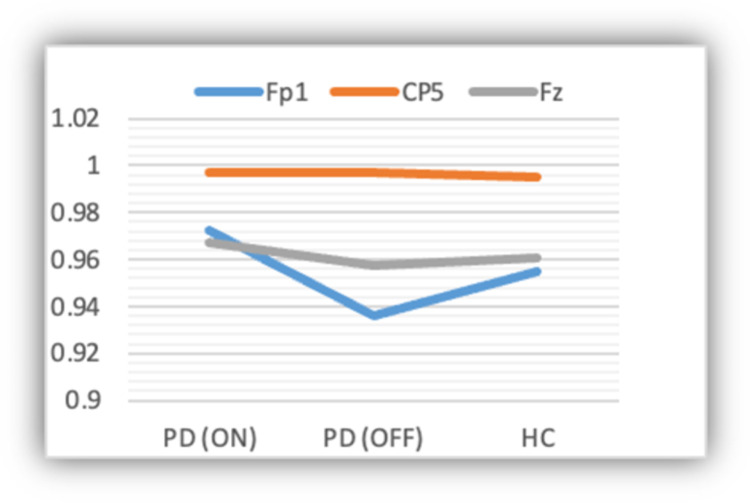
Sensitivity of the model across the three classes.

**Fig 7:**
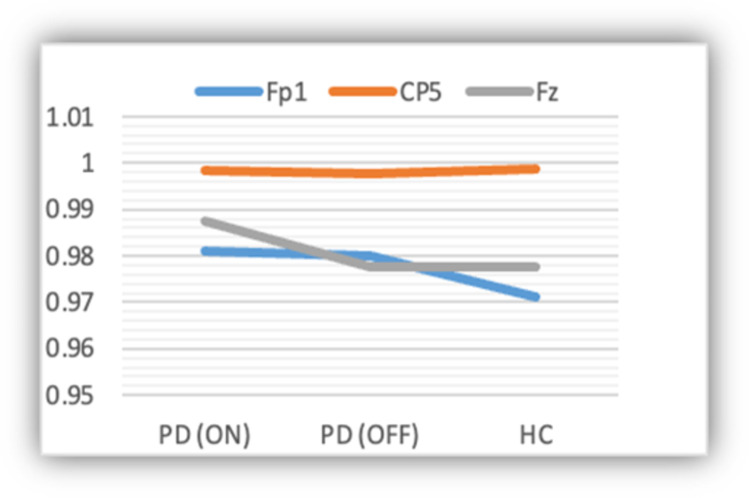
Specificity of the model across the three classes.

Figs 6 and 7 show the sensitivity and specificity of the deep-learning approach across the three different classes using the EEG data at three different channels (i.e., **Fp1**, **CP5**, and **Fp2**). Based on the figures, the approach is relatively more sensitive at **CP5**. Also, it is slightly sensitive towards PD (ON medication) as compared to the other two classes.

**Fig 8:**
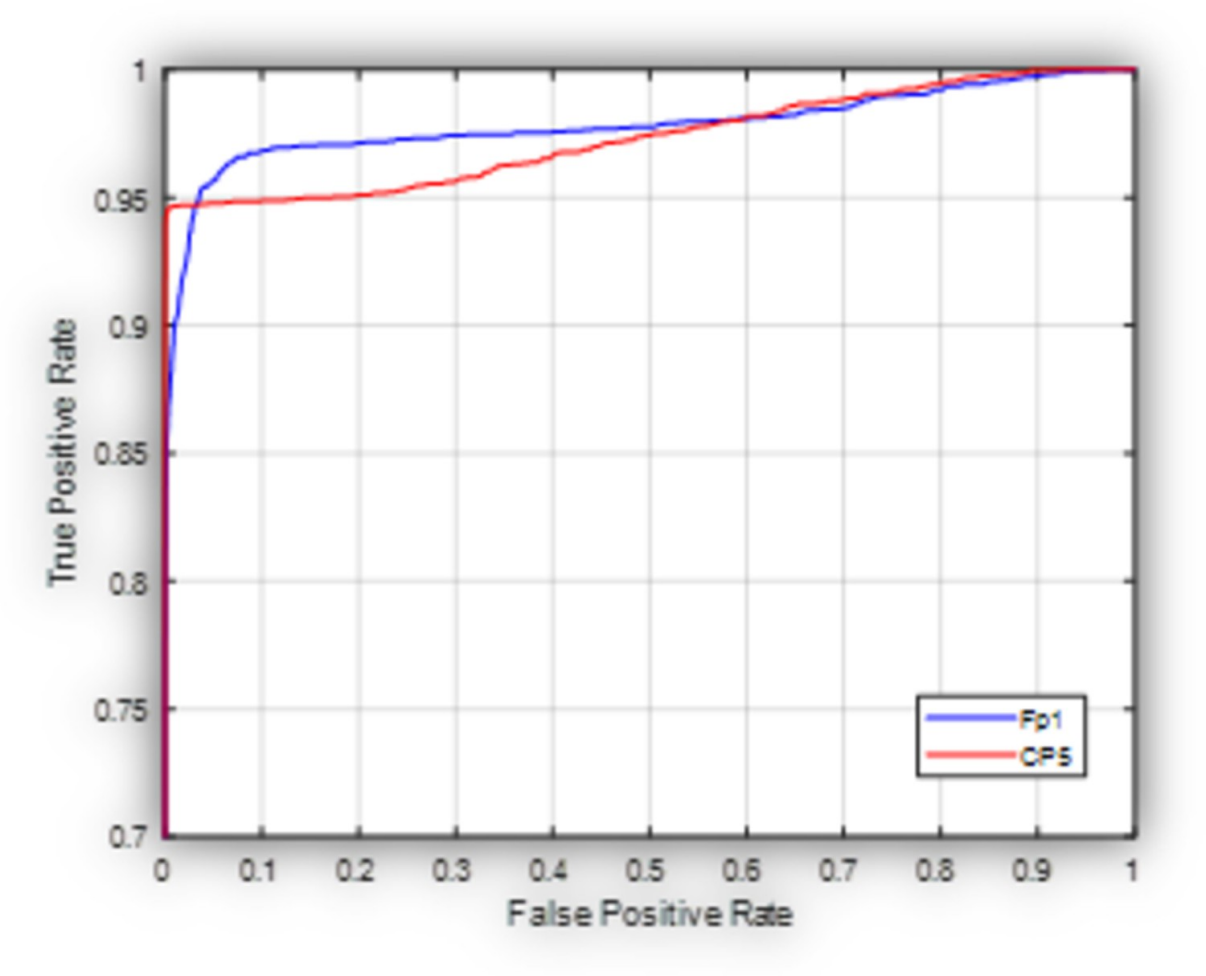
Receiver operating characteristic curve for the proposed model used to classify subjects into HC, PD (OFF medication) and PD (ON medication) at Fp1 and CP5.

Fig 8 shows the ROC of the proposed model in the generic case when it is used to classify subjects into HC, PD (OFF medication), and PD (ON medication) at Fp1 (worst case scenario) and CP5 (best case scenario) based on the 4-fold cross validation experiment. As shown in Fig 8, the proposed model exhibits a better performance at CP5 with respect to Fp1 in terms of the elevated true positive rate at a relatively low false positive rate. This may show that the values recorded at the central electrodes offer a benefit over the ones captured by frontal electrodes due to less susceptibility to eye motion artifacts as mentioned before.

In conclusion, the deep-learning approach provides a promising PD screening tool that exploits the Wavelet domain of resting-state EEG and offers a significantly accurate and sensitive decision support system for neurologists and neuroscience researchers seeking answers regarding the differentiability of PD based on resting-state EEG.

### Feature visualization using the Grad-CAM method

**Fig 9:**
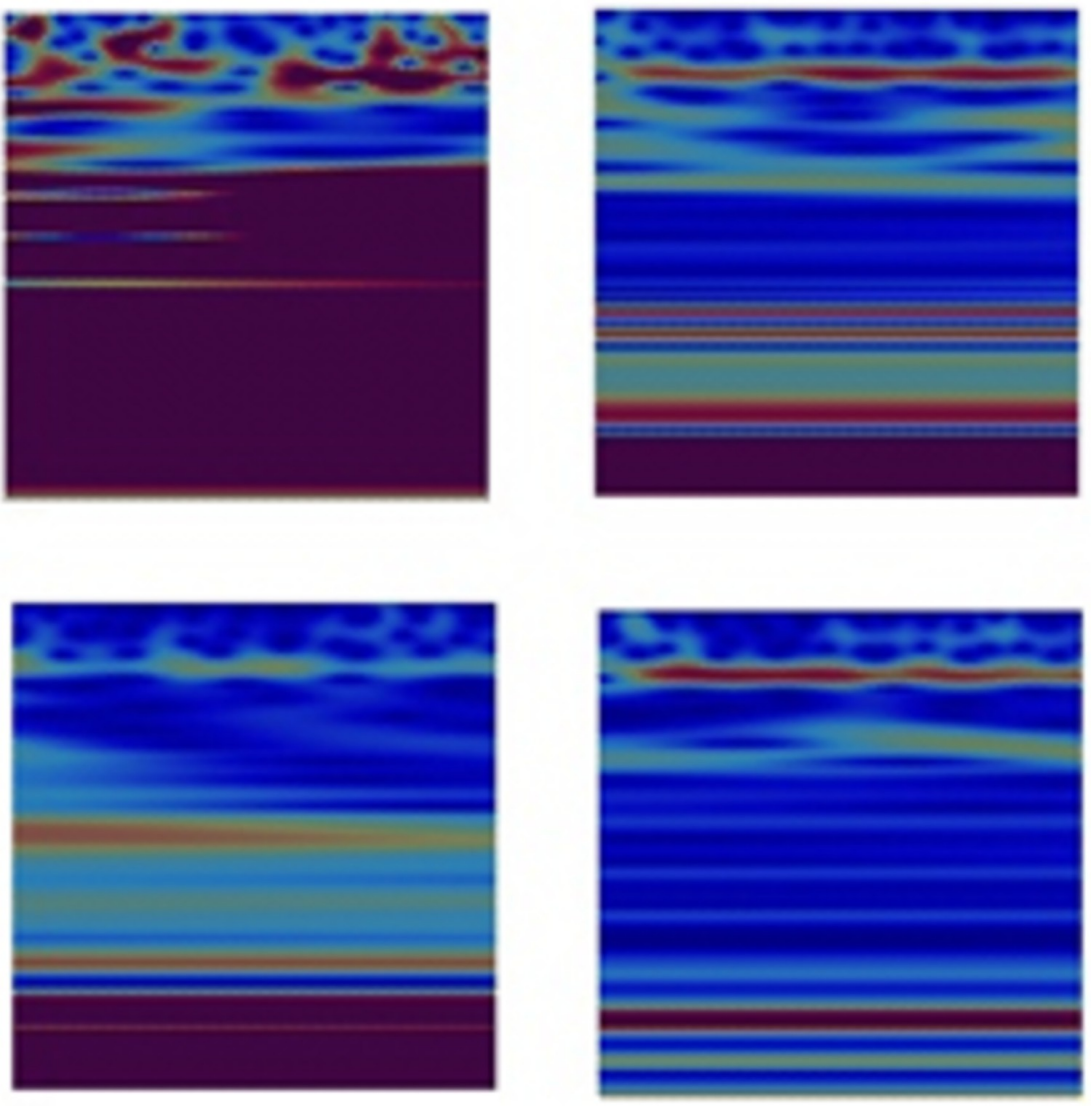
Heat maps showing the significance of the features identified in the wavelet images for HC (first column) and PD OFF medication (second column) for channels Fp1 and CP5. Channel Fp1 is represented in the first row and channel CP5 is represented in the second row.

**Fig 10:**
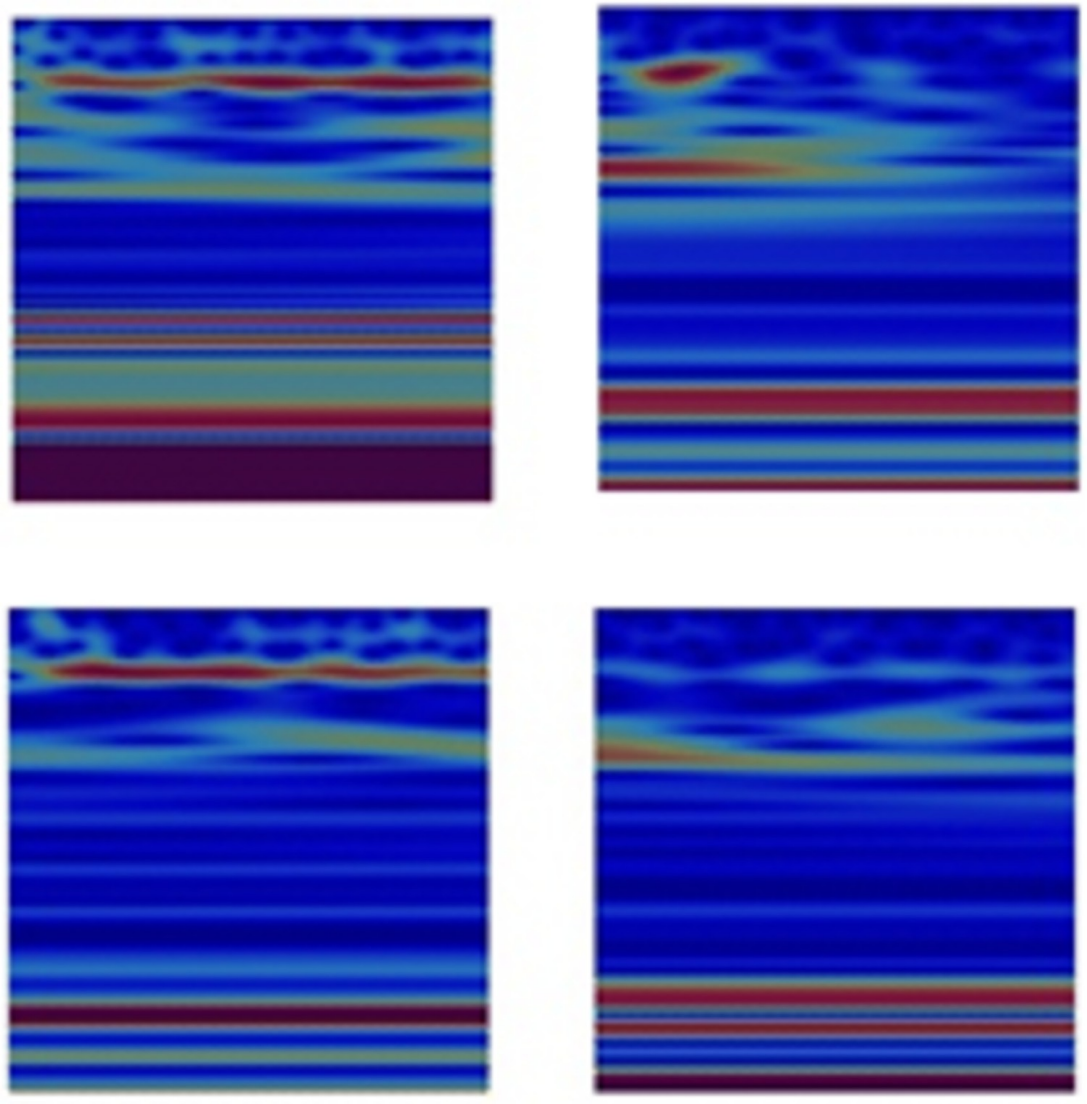
Heat maps showing the significance of the features identified in the wavelet images for PD OFF medication (first column) and PD ON medication (second column) for channels Fp1 and CP5. Channel Fp1 is represented in the first row and channel CP5 is represented in the second row.

**Fig 11:**
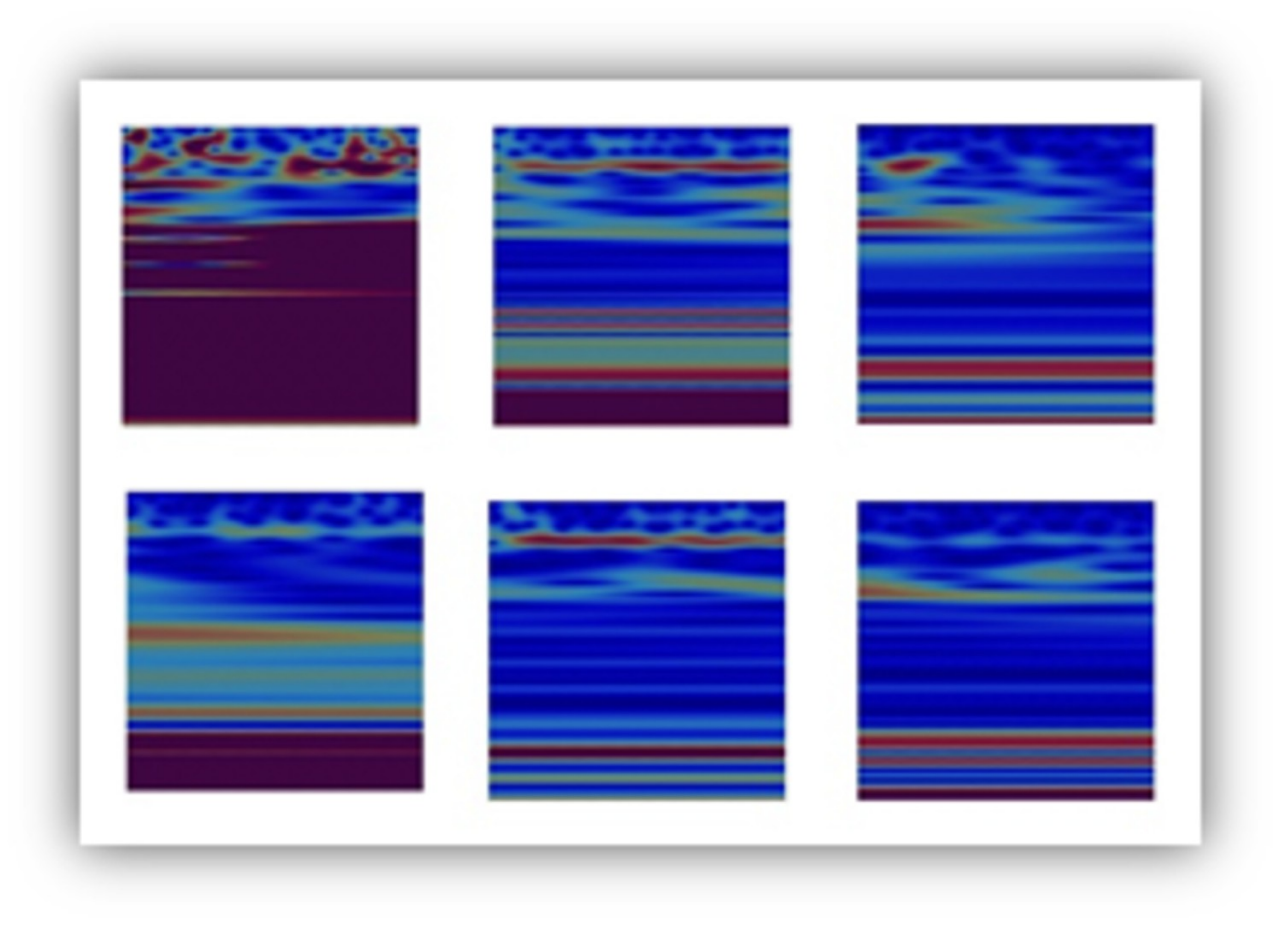
Heat maps showing the significance of the features identified in the wavelet images for HC (first column), PD OFF medication (second column) and PD ON medication (third column) for channels Fp1 and CP5. Channel Fp1 is represented in the first row and channel CP5 is represented in the second row.

In order to understand the reason behind the achieved predictions, the feature maps of the 16^th^ layer of the models used in the past three experiments were visualized using the Grad-CAM method discussed in Section III Subsection B. Figs 9–11 show the corresponding class discriminative maps for the models when validated using the EEG data at **Fp1** and **CP5**.

As shown in Fig 9, and by referring to the original Wavelets of **Fp1** and **CP5** presented in Fig 2, the deep-learning approach was able to distinguish HC from PD or PD subjects who are OFF medication based on regions of high intensity values (marked in RED) at low scales but most importantly at mid and high scales of the Wavelet images. This is more obvious in Wavelets of **Fp1** as compared to **CP5** Wavelets.

When PD (ON medication) was compared to PD (OFF medication) as in Fig 10, the features identified by the model as significant and discriminatory were the time-continuity of the high intensity values at a certain low scale range for PD (OFF medication) as compared to PD (ON medication). Further, the locations of the higher scales with relatively high intensity values were used to differentiate PD (OFF medication) from PD (ON medication) at both channels as well.

When the deep-learning approach was used in the three-class problem (i.e., classification of subjects into HC, PD (OFF medication) and PD (ON medication)), the significant feature maps used in the successful prediction of the subject status (see Fig 11) were consistent with the observations reached based on Figs 9 and 10.

### Computational time complexity

The proposed framework has achieved a significant performance in discriminating subjects with Parkinson’s Disease (PD) from healthy controls as well as the efficient classification of PD subjects into patients receiving therapeutic treatments and patients without any intervention. One of the limitations of this study is that the proposed approach has not been tested in a clinical setting or directly applied to real-time EEG being recorded for patients to provide a prompt prediction and diagnosis of the disease.

However, due to the low-complexity of the proposed approach which consists of a Continuous Wavelet transform preceded with a 20-Layer CNN, the proposed approach is expected to offer a promising real-time screening for subjects that will potentially support the clinical diagnosis of the disease. We have determined the computational time for the application of the proposed framework when applied on the offline version of the entire EEG time-series recorded at a single channel (i.e., CP5) for approximately 1.9 to 2 minutes. The EEG which was sampled at 512 S/s created a discrete-time signal of 102,400 time-samples. A computational time of 25.1 seconds was estimated on a Dell Alienware workstation with Intel(R) Core (TM) i9-9900K CPU @ 3.6GHz (8 Cores), 64 GB RAM, NVIDIA GPU using MATLAB R2021a.

### Comparative study with related work

The state-of-the-art research that adopted the use of machine and deep learning for PD diagnosis [24–29] have mostly used two different datasets (i.e., UC San Diego Resting State, and UKM Medical Center EEG datasets) while a single study [23] used a locally acquired EEG dataset at the Sun Yat-Sen University First Affiliated Hospital which is considered the largest among other datasets.

The related work adopted three different approaches:

1. Direct application of MDL techniques including hybrid CNN-RNN and ANN on the time-domain representation of EEG such as in [23–25].
2. TFR Transformation using TQWT followed with handcrafted feature extraction which is labor and computational time-intensive and then classification based on the extracted features using a machine learning technique such as LSSVM and probabilistic neural network as in [26, 29].
3. TFR Transformation using TQWT and SPWVD followed with a direct application of a deep learning technique (i.e., 2D CNN) for simultaneous feature extraction and classification as in [27, 28].

Both the second and third approach provided the highest performance in terms of validation accuracy, sensitivity and specificity as compared with the first approach as shown in Table 7. This may be due to the intrinsic discriminative features of PD that can be extracted in the TFR or time-scale domains rather than the pure time-domain. Further, the third approach with the direct application of CNN for both feature extraction and classification provides a major advantage on the second approach in terms of the reduction in the framework complexity as well as the generalization of the approach that can be directly adopted and tested on non-resting state EEG or sleep EEG without the need to validate the aforementioned handcrafted features.

**Table 7:** Comparison of the proposed approach and the-state-of-the-art architectures used for PD detection.

Approach	Dataset	TFR	MDL	Classification	Accuracy
**Shi et al., 2019 [23]**	Sun Yat-Sen University First Affiliated Hospital EEG for 40 PD and 30 HC	-	2D CNN-RNN, 3D CNN-RNN	PD Vs. HC	81.13%
					82.89%
**Lee et al., 2019 [24]**	UBC Resting State EEG for 20 PD and 22 HC	-	2D CNN-LSTM	PD Vs. HC	96.9%
**Shaban, 2021 [25]**	UC San Diego Resting State EEG for 15 PD and 16 HC	-	ANN	PD Vs. HC	98%
**Khare et al., 2021 [26]**	UC San Diego Resting State EEG for 15 PD and 16 HC	TQWT	LSSVM	HC Vs. PD (OFF Medication)	96.13%
				HC Vs. PD (ON Medication)	97.65%
**Khare et al., 2021 [27]**	1. UC San Diego Resting State EEG for 15 PD and 16 HC	SPWVD	2D CNN	HC Vs. PD (OFF Medication)	99.7%
	2. UKM Medical Center EEG for 20 PD and 20 HC			HC Vs. PD (ON Medication)	100%
**Loh et al., 2021 [28]**	UC San Diego Resting State EEG for 15 PD and 16 HC	Gabor Transform	2D CNN	HC Vs. PD (ON, OFF Medication)	99.46%
**Murugappan et al., 2020 [29]**	UKM Medical Center EEG for 20 PD and 20 HC	TQWT	Probabilistic Neural Network	PD Emotion Classification	94%
**Proposed Work**	UC San Diego Resting State EEG for 15 PD and 16 HC	CWT	2D-CNN	HC Vs. PD (OFF Medication)	**99.9%**
				PD (OFF Medication) Vs. PD (ON Medication)	99.8%
				HC Vs. PD (ON, OFF Medication)	**99.6%**

Based on the reported performance results in Table 7, the proposed CWT-CNN approach provided a cross-validation accuracy up to 99.9% at **CP5** for classifying subjects into HC and PD without medication (which is the case for the initial screening of potential PD patients) outperforming the recent state-of-the-art methods [23–27] with the highest accuracy attained by Khare et al. [27] of 99.7%. In addition, the proposed framework slightly surpassed the only framework (Gabor transform-CNN [28]) tested on a three-class challenge aimed at identifying HC from PD (ON, and OFF medication) with 0.16% improvement.

Not only the validation accuracy, sensitivity, specificity and AUC of the proposed approach reached 99.9%, but also a weighted Kappa score of 0.99 was almost achieved at **CP5** providing a clear evidence on the reliability of the performance values obtained in this study where the probability that the prediction matched the ground truth by chance is very minimum. Although the proposed approach performed slightly better at a central electrode (CP5) as compared with frontal electrodes (Fp1 and Fp2), generally the performance measures at the four selected channels were found to be comparable showing the ability of the deep-learning approach to identify the PD features regardless the location at which the EEG signal was captured.

## Discussion

PD is a complex neurodegenerative disease that is challenging for physicians and specialists to diagnose and grade. Observation of motor system abnormalities is the current means of clinical diagnosis and is the gold standard despite being subjective and prone to human error. Earlier detection of disease and initiation of neuroprotective treatments (when these are available) have potential to improve the prognosis and possibly slow down the disease progression.

In this paper, we have introduced a deep-learning approach that utilizes a recently proposed CNN structure to exploit the Wavelet domain of resting-state EEG for HC, PD (OFF medication), and PD (ON medication). The objective of this framework is to distinguish PD from HC as well as to identify the distinguishing features in EEG between PD subjects who receive therapeutic treatments and subjects without any intervention. Further, we have introduced the use of this technique on a three-class problem where deep-learning can efficiently identify normal subjects, PD (OFF medication), and PD (ON medication).

The strengths of the proposed approach can be summarized as follows: 1. The deep-learning approach was able to classify subjects into PD and HC with significantly high cross-validation accuracy, sensitivity, specificity, AUC of ROC, and Weighted Kappa Score up to 99.9% surpassing the recent state-of-the-art literature [23–28]. 2. The deep-learning framework revealed significant features of the disease where the Wavelet domain of HC (subjects without a clinical diagnosis of PD) exhibits regions of significantly high intensities at low, mid, and high scales as compared to subjects with PD. This may show neurological activity at specific EEG frequency intervals. 3. This study also demonstrated that PD (OFF medication) maintains a consistent continuous high intensity at narrow range of low-scales as compared to PD (ON medication). Further, there are significant changes in the locations of high scales that exhibit high values for both PD (OFF and ON medication).

The observations can serve as hypothesis generation for larger clinical and research studies to understand the role Parkinson’s disease plays in changing the Wavelet domain of the EEG. Although the study and the current findings are promising, the deep-learning approach has a few limitations:

1. Most of the deep-learning approaches including the proposed framework lack real-world clinical and experimental validation where the approaches are not tested on EEG data for patients awaiting clinical diagnosis. It was mentioned in [56], that the accuracy of clinical diagnosis performed by non-experts was determined as 73.8% while when the diagnosis was performed by movement disorders experts, the accuracy was found to be 79.6% (initial assessment) and 83.9% (follow-up assessment). Using AI models with accuracies that are close to 100% as attained in this study will provide a high confidence in the AI classifier predictions and will support the clinical diagnosis. In the future, we are planning to investigate the use of AI methods in sleep EEG that has been acquired for 59 patients with and without Mild Cognitive Impairment (MCI) as there have been several studies on sleep-EEG to identify potential biomarkers of PD and cognitive dysfunction [57–61]. We will further use the future developed methods to test on real-time data generated for patients and compare with expert’s annotation (i.e., ground truth). We also expect that the model will be able to run concurrently with real-time EEG data acquisition and therefore, the processing time may then slightly exceed total time required to acquire EEG.
2. Since the CWT is a highly redundant transform with a significant overlap among Wavelets at different scales, Discrete Wavelet Transform (DWT) can be deployed instead of the CWT to provide more efficient and sparse time-scale representation of EEG time-domain signals. Further, more powerful and efficient techniques including TQWT, Flexible Analytic Wavelet Transform (FAWT), and Variational Mode Decomposition (VMD) will be considered for EEG transformations prior to AI framework application as such techniques will support better identification and interpretation of the discriminative features of EEG related to each class using AI based visualization techniques.
3. EEG is not currently adopted for the clinical diagnosis of PD. However, once the EEG based deep-learning method is validated in the clinical setting and trials demonstrate the relationship between the recognized Wavelet features and PD, these EEG signatures may serve as an alternative supportive objective measure of disease status and improve the understanding of the nature of the disease, its potential EEG biomarkers, and its response to treatment.
4. Additional work is needed to determine if the proposed approach can effectively serve as a screening method to identify subjects with high risk to develop PD, since the current approach was trained and validated on a dataset for subjects with a confirmed PD diagnosis. Pre-clinical diagnosis of PD may help improve the efficacy of the therapeutic treatment and potentially delay the progression of the disease. Subjects with prodromal PD (such as those with REM sleep behavior disorder) will be an ideal population in which to test this.

## Abbreviations

CNN: Convolutional Neural Networks
DL: Deep-Learning
Grad-CAM: Gradient-Weighted Class Activation Mapping
PD: Parkinson’s Disease
